# Integrative Microbiome–Metabolome Analysis Reveals Dose-Related Effects of Rumen-Derived *Ligilactobacillus salivarius* KS1018 on Rumen Function in Lambs

**DOI:** 10.3390/microorganisms14081641

**Published:** 2026-07-27

**Authors:** Hui Jiang, Zixuan Wang, Shiyu Duan, Cong Peng, Yuchen Jia, Xiyue Deng, Yiping Zhu, Jing Li

**Affiliations:** 1 College of Veterinary Medicine, China Agricultural University, Beijing 100193, China; jhcauvet@163.com (H.J.); zixuanw@cau.edu.cn (Z.W.); daunsyvet@cau.edu.cn (S.D.); sszspc1023@163.com (C.P.); jiayuchen@cau.edu.cn (Y.J.); 2021317010324@cau.edu.cn (X.D.); 2 State Key Laboratory of Veterinary Public Health and Safety, China Agricultural University, Beijing 100193, China

**Keywords:** rumen-derived *Ligilactobacillus salivarius*, rumen microbiota, rumen metabolome, rumen function

## Abstract

Rumen-derived probiotics may help regulate fermentation and microbial activity in lambs, but the dose-related effects remain unclear. This study evaluated the effects of rumen-derived *Ligilactobacillus salivarius* KS1018 on rumen function in Hu lambs using integrated assessments of growth performance, serum hormones, rumen fermentation, rumen microbiota and metabolomics profiling. Thirty-two male Hu lambs were allocated to four groups (*n* = 8/group): control (C) or KS1018 at 0.5 × 10^9^ (L), 1.0 × 10^9^ (M), or 1.5 × 10^9^ CFU/lamb/day (H) for 56 days. Growth performance, including final BW and ADG, did not differ significantly among groups (*p* > 0.05). Serum T3 and T4 were significantly higher in M and H than in C (*p* < 0.01). Group H exhibited significantly lower NH_3_-N and higher total VFA concentration of rumen fluid compared with group C (*p* < 0.05). Significant increases in isobutyric acid concentration were observed in groups M and H (*p* < 0.05). Genus-level shifts and dose-related metabolite changes were detected; oxidative phosphorylation was enriched in group H and amino acid biosynthesis in group M. Procrustes analysis indicated significant concordance between the rumen microbiome and metabolome datasets (*M*^2^ = 0.56, *p* < 0.001). These results indicate that KS1018 supplementation was associated with dose-related changes in rumen fermentation and selected microbiome–metabolome associations in Hu lambs, although the optimal dose requires further validation.

## 1. Introduction

The rumen microbiome plays a central role in ruminant nutrition by converting plant fibers into volatile fatty acids (VFAs), directly influencing host health and productivity [1,2]. To comprehensively evaluate rumen function, it is informative to integrate fermentation measurements with rumen microbiome profiling and rumen metabolomics, as these approaches provide complementary information on fermentation outputs, microbial community structure, and metabolic activity, respectively. In this context, VFA profiles, ruminal ammonia nitrogen (NH_3_-N) and ruminal pH are widely used practical indicators of fermentation status and potential metabolic risk [3], while microbiome and metabolome profiles help characterize the underlying microbial and biochemical shifts associated with these readouts [4].

Probiotics have been used as a sustainable strategy to support rumen function by promoting fiber digestion, maintaining microbial balance, and inhibiting the overgrowth of pathogens such as *Streptococcus bovis* [5]. They have also been associated with improved feed efficiency and a reduced risk of fermentation disorders such as acidosis [6,7]. In some studies, specific strains such as *Ruminococcus albus* and *Ruminococcus flavefaciens* have been reported to promote the enrichment of cellulose-degrading bacteria, leading to increased VFA production and reduced ammonia nitrogen (NH_3_-N) levels [6,8]. Other work suggests that probiotics help maintain a favorable anaerobic rumen environment that supports beneficial microbes, optimizing fermentation efficiency and stabilizing rumen pH [9,10]. Current research on ruminant probiotics has included yeast supplements for fiber digestion [11] and bacterial blends for immune modulation [12]. Together, these findings support the potential of probiotics to influence fermentation outputs and microbial activity, and to modulate rumen metabolite profiles.

*Ligilactobacillus salivarius* is a key bacterium isolated from many species, demonstrating critical probiotic properties. In poultry, *L. salivarius* CTC2197 have demonstrated the ability to prevent *Salmonella enteritidis* colonization by secreting antimicrobial substances and inhibiting pathogen adhesion [13,14]. In pigs, supplementation with *L. salivarius* reduces diarrhea and improves gut health by increasing lactic acid bacteria and inhibiting pathogenic *E. coli* [15]. In ruminants, *L. salivarius* has been shown to improve growth, gut health, and immunity [16,17]. In yak calves, supplementation with *L. salivarius* reduced diarrhea and promoted weight gain while positively modulating intestinal microbiota [16]. In Murrah buffalo calves, a probiotic consortium containing *L. salivarius* improved growth performance, feed efficiency, and beneficial gut bacteria [17].

Previous research by our team identified *Ligilactobacillus salivarius* KS1018 as a host-derived probiotic candidate and demonstrated that supplementation in Hu sheep improved immune function and antioxidant capacity, with concomitant changes in metabolic indicators and gastrointestinal histology, while maintaining overall stability of the fecal microbiota [18]. These results highlight its beneficial effects at the host level, but its impact on rumen microbial ecology and fermentation remains unclear. This study aims to investigate the potential probiotic effects of *L. salivarius* KS1018 by conducting feeding trials in Hu lambs. Growth-related outcomes and key rumen fermentation parameters were compared, including VFA profiles, ruminal ammonia nitrogen (NH_3_-N) and ruminal pH, which are closely linked to feed utilization and nitrogen metabolism. In addition, rumen microbiome profiling and rumen metabolomics were conducted to describe microbial community shifts and metabolite changes associated with fermentation responses, and to examine their relationships with major fermentation profiles.

## 2. Materials and Methods

### 2.1. Animal Ethics

The study was approved by the Animal Welfare and Ethics Committee of China Agricultural University (Approval No. AW00213202-2-2). Written consent was obtained from the farm for animal participation.

### 2.2. Preparation of Ligilactobacillus salivarius KS1018

The strain *L. salivarius* KS1018 was isolated from the rumen fluid of healthy Duolang sheep in Kashgar, Xinjiang. In vitro evaluations of probiotic properties, including stress tolerance, antimicrobial activity, and biochemical characteristics, were conducted previously [19]. For the feeding trial, *L. salivarius* KS1018 was prepared as a freeze-dried powder (≥1.0 × 10^9^ CFU/g) by Shandong Dingchuang Biotechnology (Dongying, China) using a fed-batch fermentation process. The strain was activated and then inoculated into a 100 L fermenter (60% working volume). Dissolved oxygen was kept at 30% by automatically adjusting agitation (200–600 rpm) and aeration (1.0–1.2 vvm). After fermentation, the cells were collected by disk stack centrifugation, pre-frozen at −45 °C and freeze-dried, and milled into 80–100 mesh powder, resulting in a final viable count of 7.6 × 10^9^ CFU/g.

### 2.3. Experimental Design and Animal Management

A total of 32 male Hu lambs (105 days old, 30 ± 4 kg) were randomly assigned to four groups (*n* = 8 per group): a control group (C), and three experimental groups designated as low-dose (L: 0.5 × 10^9^ CFU/lamb/day), medium-dose (M: 1.0 × 10^9^ CFU/lamb/day), and high-dose (H: 1.5 × 10^9^ CFU/lamb/day) *L. salivarius* supplementation were individually administered to each lamb by mixing the designated dose into the morning concentrated feed. All lambs had ad libitum access to roughage and water throughout the 56-day trial period. During the trial, one lamb in group C developed respiratory signs on day 20 and was found dead on day 21 after receiving supportive fluid therapy and antibiotic treatment. Based on the clinical presentation and gross post-mortem examination, the death was attributed to ovine respiratory complex, a common respiratory disease problem in intensive sheep farms in this region. The clinical signs were consistent with typical ORC manifestations, including respiratory distress, nasal discharge, cough, fever, depression. Because this lamb was in the control group and did not receive KS1018 supplementation, the death was considered unrelated to KS1018 administration. Consequently, seven lambs in group C and eight lambs in each supplemented group remained available for endpoint sampling, resulting in a total of 31 lambs.

Pre-trial procedures included deworming, individual identification, and daily health monitoring. Feed samples collected at the beginning of the trial, as well as on day 28 and 56 were pooled for compositional analysis, the analyzed nutrient composition is presented in Table S1.

### 2.4. Growth Performance Assessment

Fasting body weight (BW) was recorded on days 1, 28, and 56 of the trial. Average daily gain (ADG) was calculated for each phase, including day 1 to day 28, day 28 to day 56, and the overall study period. Feed intake and feed residue per group were measured throughout the day on days 1, 28, and 56 to calculate average daily feed intake (ADFI) per lamb throughout the trial. Feed conversion ratio (FCR) was derived from ADG and ADFI. In addition, one lamb in group M developed ovine respiratory complex (ORC) and received therapeutic treatment with tylosin, lincomycin, and Maxing Shigan injection during the trial. Because the disease and treatment could have affected feed intake and body-weight gain, this lamb was excluded from the growth-performance analysis but remained included in endpoint blood and rumen fluid sampling. Thus, growth-performance analysis included seven lambs in groups C and M and eight lambs in groups L and H.

### 2.5. Serum Hormonal Profiling

At the end of the feeding trial, blood samples (10 mL) were collected from the jugular vein of all lambs in heparinized tubes before morning feeding. Plasma was separated by centrifugation (3000× *g*, 15 min) and stored at −20 °C for further analysis. Hormones including triiodothyronine (T3), thyroxine (T4), and growth hormone (GH), were quantified using ELISA kits (Meimian, Yancheng, China).

### 2.6. Rumen Fluid Collection and Fermentation Parameter Analysis

30 mL rumen fluid was collected from each lamb via orogastric intubation before morning feeding. The first 15 mL was discarded to minimize saliva contamination [20]. The remaining fluid was filtered through four layers of gauze, and the pH was measured immediately using a portable pH meter (Sanliang PH100, Dongguan, China).

20 mL of strained rumen fluid was stored at −20 °C for ammonia nitrogen (NH_3_-N) and volatile fatty acid (VFA) determination, and an additional 10 mL was stored at −80 °C for rumen microbiome and metabolomics analyses. The NH_3_-N concentration was measured by steam distillation followed by titration with dilute hydrochloric acid, adapted from previously published manual distillation and titration procedures with minor modifications [21,22]. VFA concentrations were determined by gas chromatography based on published extraction and GC-based methods for rumen organic acids, with minor adjustments to match our sample handling and instrument settings [23].

### 2.7. Statistical Analysis of Growth Performance, Serum Hormones, and Rumen Parameters

Data are presented as mean ± SD. Repeated BW measurements were analyzed using a linear mixed-effects model in R 4.6.0 with the nlme package (version 3.1-169). Treatment, time, and their interaction were fixed effects, with animal as a random intercept. Models were fitted by maximum likelihood and evaluated using likelihood-ratio tests. ADG was analyzed using ordinary one-way ANOVA, whereas ADFI and FCR were presented descriptively because feed intake was recorded at the group level. Normality of model residuals was assessed using the Shapiro–Wilk test, and homogeneity of variance was assessed using the Brown–Forsythe test. Serum hormone concentrations and rumen fermentation parameters were analyzed using ordinary one-way ANOVA followed by Tukey’s multiple-comparisons test in GraphPad Prism 10.1.2. Kruskal–Wallis tests were additionally conducted as sensitivity analyses for variables showing evidence of non-normal residuals, including GH, T3, T4, propionic acid, and isocaproic acid. Isobutyric acid was also analyzed using Kruskal–Wallis because of its high within-group variability. The results of these sensitivity analyses were compared with the ANOVA conclusions. For rumen fermentation parameters, linear trends across increasing KS1018 doses were tested using dose levels coded as 0, 0.5, 1.0, and 1.5 × 10^9^ CFU/lamb/day for groups C, L, M, and H, respectively. Significance was set at *p* < 0.05.

### 2.8. DNA Extraction and 16S rRNA Gene Sequencing of Ruminal Microbiota

Total microbial genomic DNA was extracted from rumen fluid samples using the FastPure Stool DNA Kit (MJYH Biotechnology, Shanghai, China). The extracted DNA was verified via electrophoresis and quantified using a NanoDrop ND-2000 spectrophotometer (Thermo Fisher Scientific, Waltham, MA, USA). The V3–V4 region of the 16S rRNA gene was amplified using the primers 338F (5′-ACTCCTACGGGAGGCAGCA-3′) and 806R (5′-GGACTACHVGGGTWTCTAAT-3′) [24] under the following PCR conditions: initial denaturation at 95 °C for 3 min, followed by 27 cycles of 95 °C for 30 s, 55 °C for 30 s, and 72 °C for 45 s, with a final extension at 72 °C for 10 min. Purified amplicons were sequenced on an Illumina NextSeq 2000 platform (Majorbio Bio-Pharm, Shanghai, China) using paired-end 300 bp reads.

### 2.9. Bioinformatics and Statistical Analysis

Operational taxonomic units (OTUs) were clustered, and rarefaction curves, as well as alpha diversity indices (including observed OTUs, Chao1 richness, and Shannon index), were analyzed using QIIME 2 (v.2020.6) for each group. Differences in microbial diversity among groups were assessed using the Kruskal–Wallis test (*p* < 0.05). Community barplots were generated to visualize OTU distribution at different taxonomic levels. To evaluate beta diversity, principal coordinate analysis (PCoA) was conducted based on Bray–Curtis dissimilarity, and group differences were statistically tested using ANOSIM with 999 permutations. Linear discriminant analysis (LDA) effect size (LEfSe) was performed to identify significantly enriched bacterial taxa (from phylum to genus-level) among groups, with an LDA score > 2.5 and *p* < 0.05.

### 2.10. Rumen Fluid Metabolomic Profiling with Metabolite Extraction and Quality Control

Metabolites were extracted by adding 100 µL of rumen fluid to 400 µL of ice-cold acetonitrile:methanol (1:1, *v*/*v*) containing 0.02 mg/mL L-2-chlorophenylalanine as an internal standard. The mixture was vortexed for 30 s, sonicated at 40 kHz at 5 °C for 30 min, and then centrifuged. After protein precipitation at −20 °C for 30 min and nitrogen drying, the residues were reconstituted in acetonitrile:water (1:1, *v*/*v*), sonicated at 5 °C for 5 min, and centrifuged before liquid chromatography–mass spectrometry (LC-MS) analysis. A pooled quality control (QC) sample, consisting of equal volumes from all experimental samples, was prepared and analyzed at five-sample intervals to ensure system stability and reproducibility.

### 2.11. LC-MS/MS Analysis and Data Processing

Metabolite profiling was conducted using a SCIEX UPLC-Triple TOF 6600 system (Majorbio Bio-Pharm, China) equipped with an ACQUITY HSS T3 column (100 × 2.1 mm, 1.8 μm). The mobile phase consisted of solvent A (0.1% formic acid in water:acetonitrile, 95:5) and solvent B (0.1% formic acid in acetonitrile:isopropanol:water, 47.5:47.5). Gradient elution was applied as follows: positive ion mode (0–8 min, 0–100% B) and negative ion mode (0–8 min, 0–100% B). The flow rate was set at 0.40 mL/min, with a column temperature of 40 °C. For mass spectrometry (MS) analysis, electrospray ionization (ESI) was performed at ±5000 V, with a source temperature of 550 °C, curtain gas pressure of 30 psi, and collision energy ranging from 20 to 60 eV. Data acquisition was carried out in Information Dependent Acquisition (IDA) mode across an *m*/*z* range of 50–1000.

Raw data were processed using Progenesis QI software (v3.1, Waters, Milford, MA, USA) for baseline correction, peak alignment, and retention time calibration. Metabolite annotation was performed using the Human Metabolome Database (HMDB), Metlin, and the Majorbio Database (MJDB). Data matrices were filtered to retain features detected in ≥80% of samples, normalized via sum normalization, and subjected to quality control (QC)-based exclusion (RSD > 30%). Multivariate statistical analyses, including principal component analysis (PCA) and orthogonal partial least squares discriminant analysis (OPLS-DA), were conducted using the R package “ropls” (v1.6.2). For pairwise comparisons, differential metabolites were screened based on VIP > 1 and *p* < 0.05. For multi-group comparisons, one-way ANOVA followed by Scheffe’s post hoc test was used. FDR-adjusted q values were calculated to account for multiple testing and are reported in Table S2. KEGG pathway enrichment and functional annotation were performed using the Majorbio Cloud Platform (cloud.majorbio.com), with statistical analysis conducted via Python’s “scipy.stats”(v1.18.0) and referenced against the KEGG database. For graphical presentation, metabolism-related KEGG pathways were prioritized to focus on pathways with clearer relevance to rumen metabolomic interpretation. Metabolite annotations were putative and were based on accurate mass and database-assisted MS/MS fragmentation matching. Authentic standards were not analyzed; therefore, none of the annotations were considered MSI level 1. Annotation confidence, fragmentation scores, mass errors, and database identifiers are provided in Table S3.

### 2.12. Rumen Microbiome–Metabolome Interaction Analysis

Procrustes analysis was performed to compare the microbiome and metabolome datasets. Mantel tests were performed separately within each treatment group to assess correlations between the genus-level microbial community distance matrix and individual metabolite distance matrices. Bray–Curtis distances were calculated for both microbial abundance and metabolite data, and correlations were evaluated using Spearman’s method.

A correlation heatmap was generated to visualize the interactions between the microbiome and metabolome. The microbial feature set consisted of the top 20 genus-level taxa with significant differences among groups. The metabolite set included the top 20 differential metabolites selected based on abundance and statistical significance in the multi-group comparison. Spearman correlation was applied to examine the relationships, and clustering was performed using the complete linkage method.

## 3. Results

### 3.1. Growth-Performance Analysis

Growth-performance analysis included seven lambs in groups C and M and eight lambs in groups L and H. Body weight increased significantly over time (χ^2^(2) = 195.03, *p* < 0.001). However, neither the treatment effect (χ^2^(3) = 2.87, *p* = 0.412) nor the treatment × time interaction (χ^2^(6) = 3.72, *p* = 0.714) were significant, indicating that KS1018 supplementation did not significantly alter body-weight trajectories during the 56-day trial. ADG did not differ significantly among groups during any experimental period (*p* > 0.05; Table 1). ADFI and FCR were presented descriptively. Overall, growth performance did not differ among groups at the tested doses.

**Figure 1:**
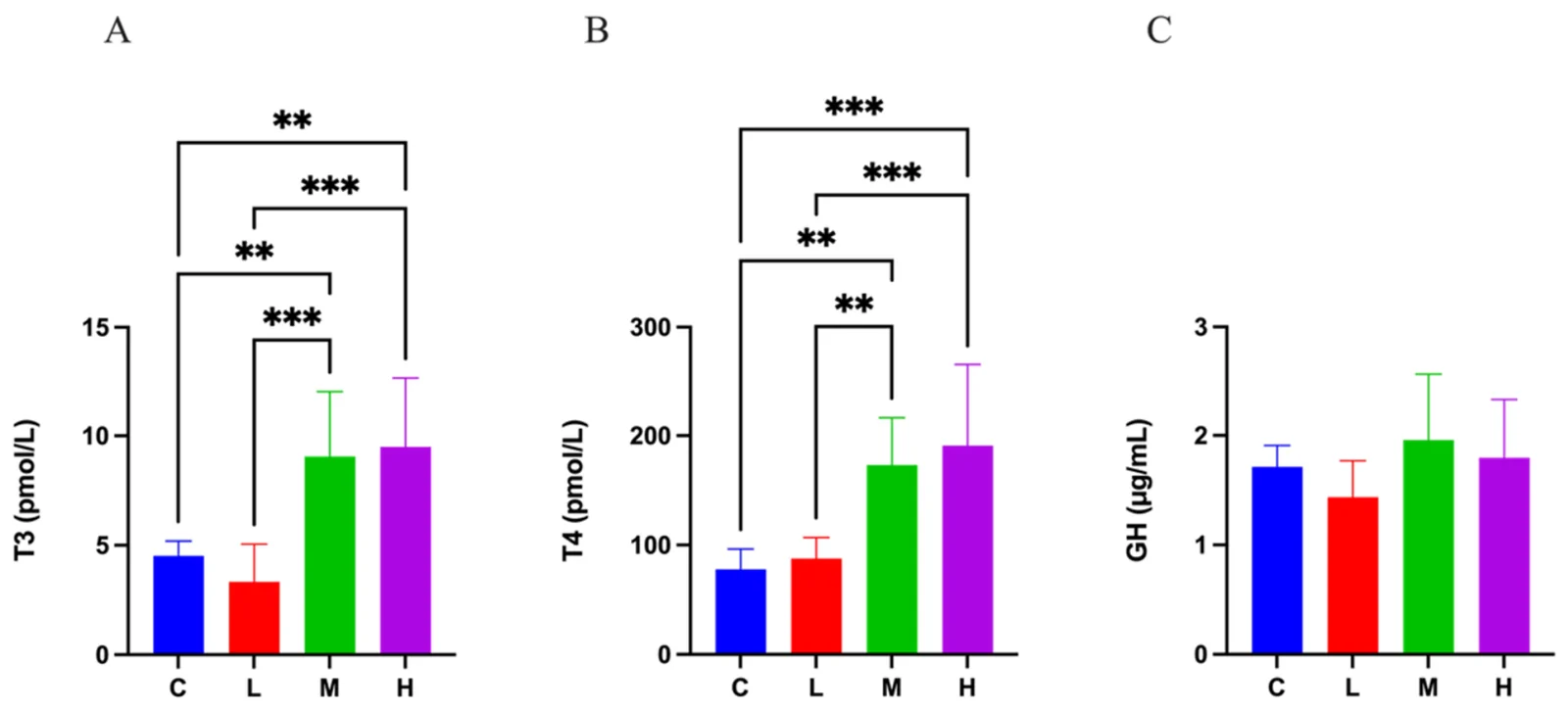
Serum thyroid hormones of lambs supplemented with *L. salivarius* during a 56-day trial. (**A**) Triiodothyronine (T3), (**B**) thyroxine (T4), (**C**) growth hormone (GH). ** *p* < 0.01, *** *p* < 0.001. Notes: C: control group; L: low-dose *L. salivarius* group; M: medium-dose *L. salivarius* group; H: high-dose *L. salivarius* group. In all subsequent figures, the abbreviations are used consistently to represent the different experimental groups.

Serum T3 and T4 were significantly elevated in groups M and H compared to group C (*p* < 0.01, Figure 1A,B). Specifically, group M exhibited a 100.4% increase in T3 (9.06 ± 2.97 pmol/L vs. 4.5 ± 0.68 pmol/L) and a 98.7% increase in T4 (173.0 ± 43.29 pmol/L vs. 77.99 ± 18.44 pmol/L). Similarly, group H showed a 100.4% increase in T3 (9.49 ± 3.15 pmol/L vs. 4.52 ± 0.68 pmol/L) and a 104.1% increase in T4 (191.0 ± 74.13 pmol/L vs. 77.99 ± 18.44 pmol/L). In contrast, serum GH levels showed no significant differences among groups M, H, and C (*p* > 0.05, Figure 1C). Kruskal–Wallis sensitivity analyses were performed for GH, T3, and T4, and the results were consistent with the ANOVA conclusions.

### 3.2. Rumen Fermentation Characteristics

**Figure 2:**
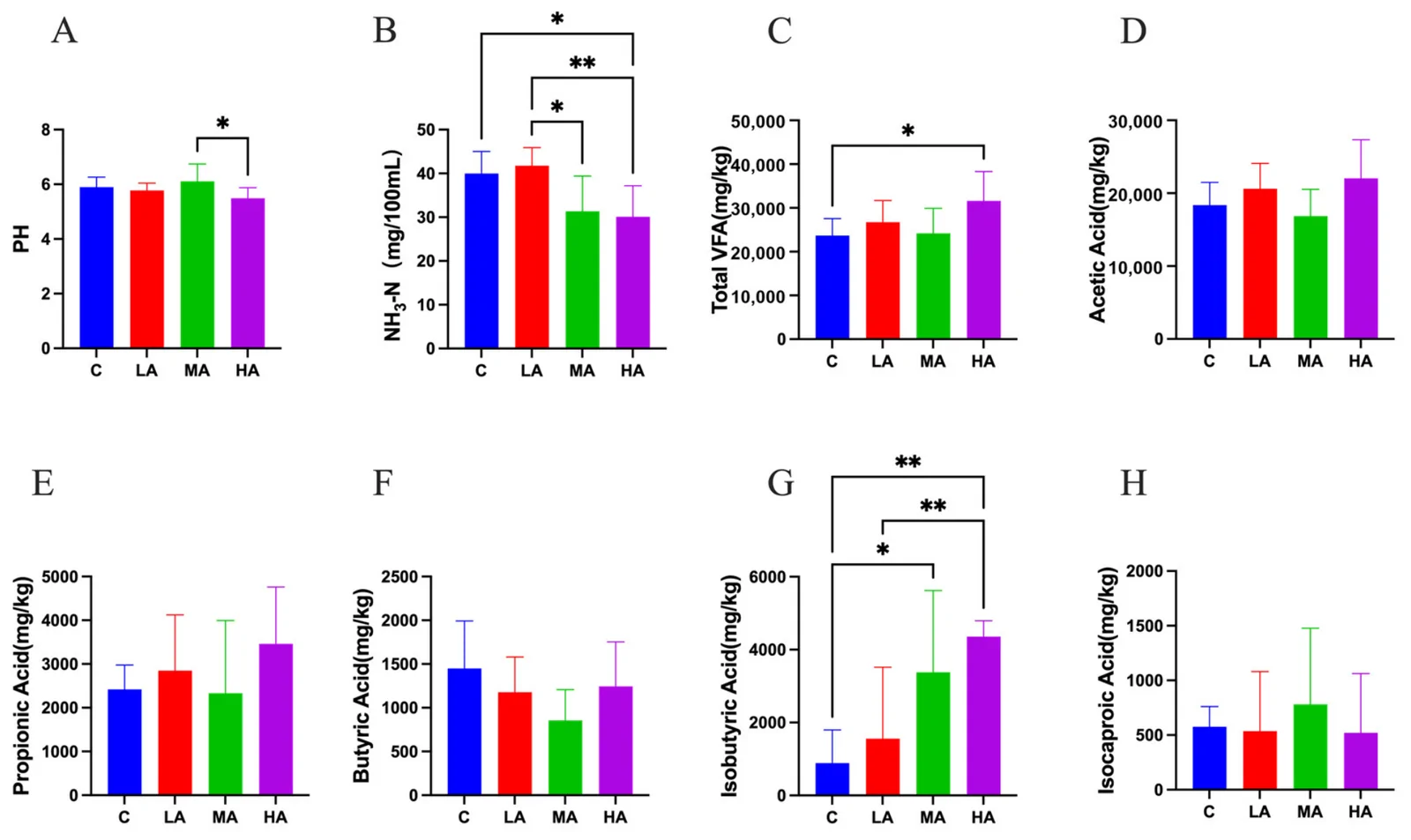
Impact on rumen fermentation and nitrogen metabolism supplemented with *L. salivarius* KS1018 in Hu lambs. (**A**) pH, (**B**) NH_3_-N, (**C**) total VFA, (**D**) acetic acid, (**E**) propionic acid, (**F**) butyric acid, (**G**) isobutyric acid, (**H**) isocaproic acid. * *p* < 0.05, ** *p* < 0.01.

*L. salivarius* supplementation significantly influenced rumen nitrogen metabolism and VFA profiles (Figure 2). The pH of group M was significantly higher than that of group H (M vs. H: 6.11 ± 0.63 vs. 5.49 ± 0.39, *p* = 0.0262, Figure 2A), although no significant linear trend was observed across increasing KS1018 doses (*p*-linear = 0.216). Ruminal ammonia nitrogen (NH_3_-N) concentration was significantly affected by treatment, with group H showing a significant decrease compared with group C (*p* = 0.027, Figure 2B). Furthermore, both group M and group H exhibited lower NH_3_-N levels than group L (M vs. L: *p* = 0.013; H vs. L: *p* = 0.005, Figure 2B). Linear trend analysis further showed that NH_3_-N concentration decreased significantly with increasing KS1018 dose (*p*-linear < 0.001), indicating a dose-related reduction in ruminal ammonia accumulation.

Total VFA concentration was significantly higher in group H compared with group C (*p* = 0.043, Figure 2C), and showed a significant positive linear trend across increasing KS1018 doses (*p*-linear = 0.024). For specific VFAs, butyric acid levels showed a tendency to decrease in group M compared with group C (*p* = 0.078, Figure 2F), but no significant linear trend was detected (*p*-linear = 0.217). Isobutyric acid levels were significantly higher in both group M and group H (M vs. C: *p* = 0.026; H vs. L: *p* = 0.001, Figure 2G), and increased linearly with increasing KS1018 dose (*p*-linear < 0.001). These results suggest that KS1018 supplementation produced dose-related changes in rumen fermentation, mainly characterized by decreased NH_3_-N and increased total VFA and isobutyric acid concentrations. Kruskal–Wallis sensitivity analyses were performed for propionic acid, isocaproic acid, and isobutyric acid, and the results were consistent with the ANOVA conclusions; notably, isobutyric acid remained significantly different among groups (*p* = 0.018), supporting the robustness of this result despite high within-group variability.

The effects of *L. salivarius* KS1018 supplementation on rumen fermentation parameters are summarized in Table 2.

### 3.3. Ruminal Microbiota Diversity and Composition Analysis Supplemented with L. salivarius KS1018

**Figure 3:**
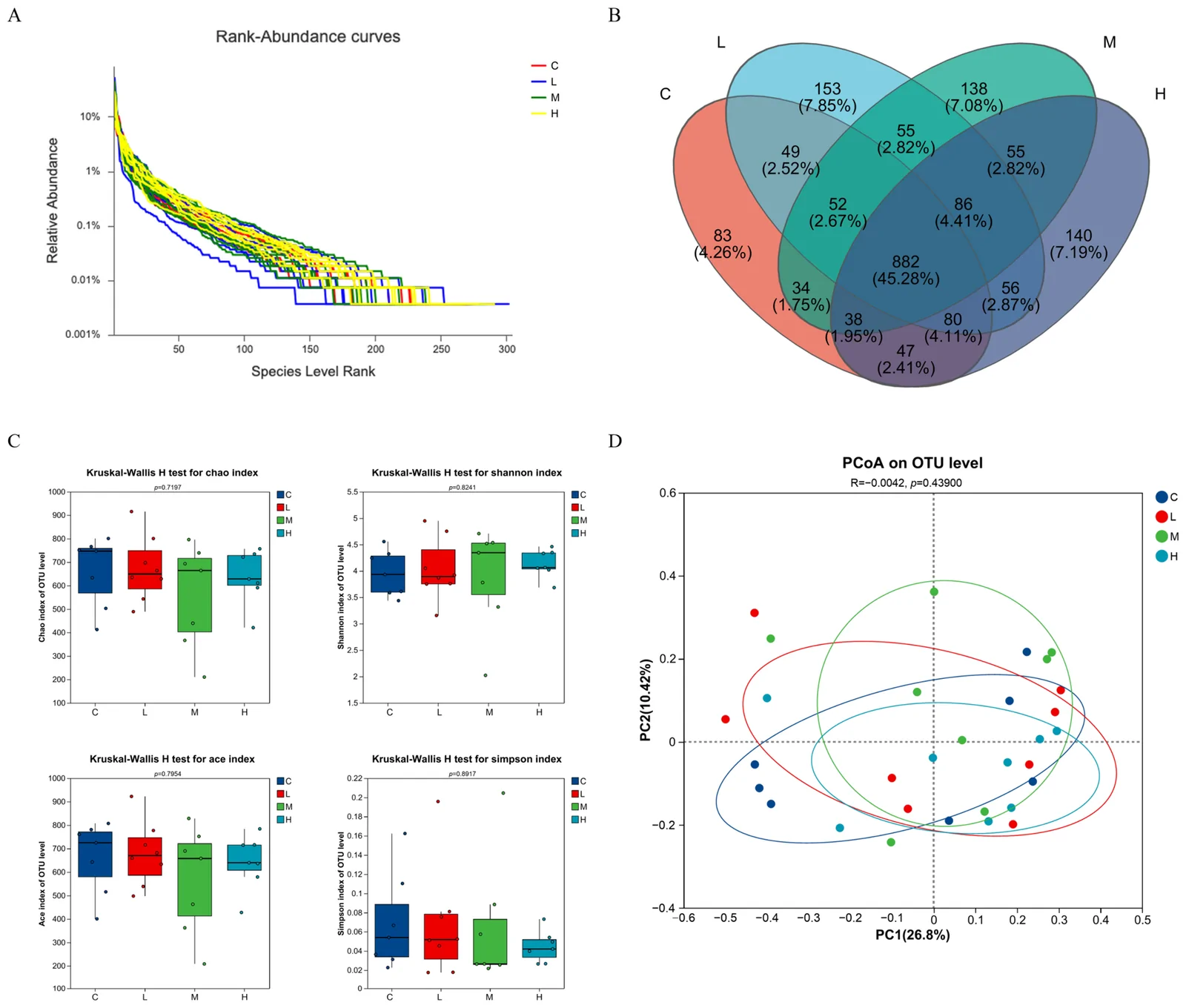
Rumen microbiota diversity and community composition in lambs supplemented with *L. salivarius* KS1018. (**A**) Rarefaction curves, (**B**) venn diagram, (**C**) alpha diversity indices (Shannon, Chao1, ACE, Simpson), (**D**) principal coordinate analysis (PCoA) plot.

Rumen fluid was available from 31 animals for microbiome analysis (C, *n* = 7; L, M, and H, *n* = 8 each). A total of 1,381,641 effective reads were obtained after quality control, with an average of 44,579 reads per sample. As shown in Figure 3A, the rarefaction curves indicated that there were sufficient microbial sequences to accurately describe the ruminal bacterial composition of each group. Based on 97% similarity, 1939 OTUs were annotated, of which 882 OTUs were shared across all groups (Figure 3B).

Compared to group C, lambs from groups L, M, and H showed no significant differences in the alpha diversity indices (Shannon, Chao1, ACE, and Simpson) in the rumen (Figure 3C). Principal coordinate analysis (PCoA) revealed that PCo1 and PCo2 explained 26.8% and 10.42% of the variation among samples, respectively (Figure 3D). The plots for the different groups displayed considerable overlap, indicating minimal divergence in bacterial community composition. The bacterial community composition did not show significant differences among group C and groups L, M, H (R = −0.0042, *p* = 0.439).

**Figure 4:**
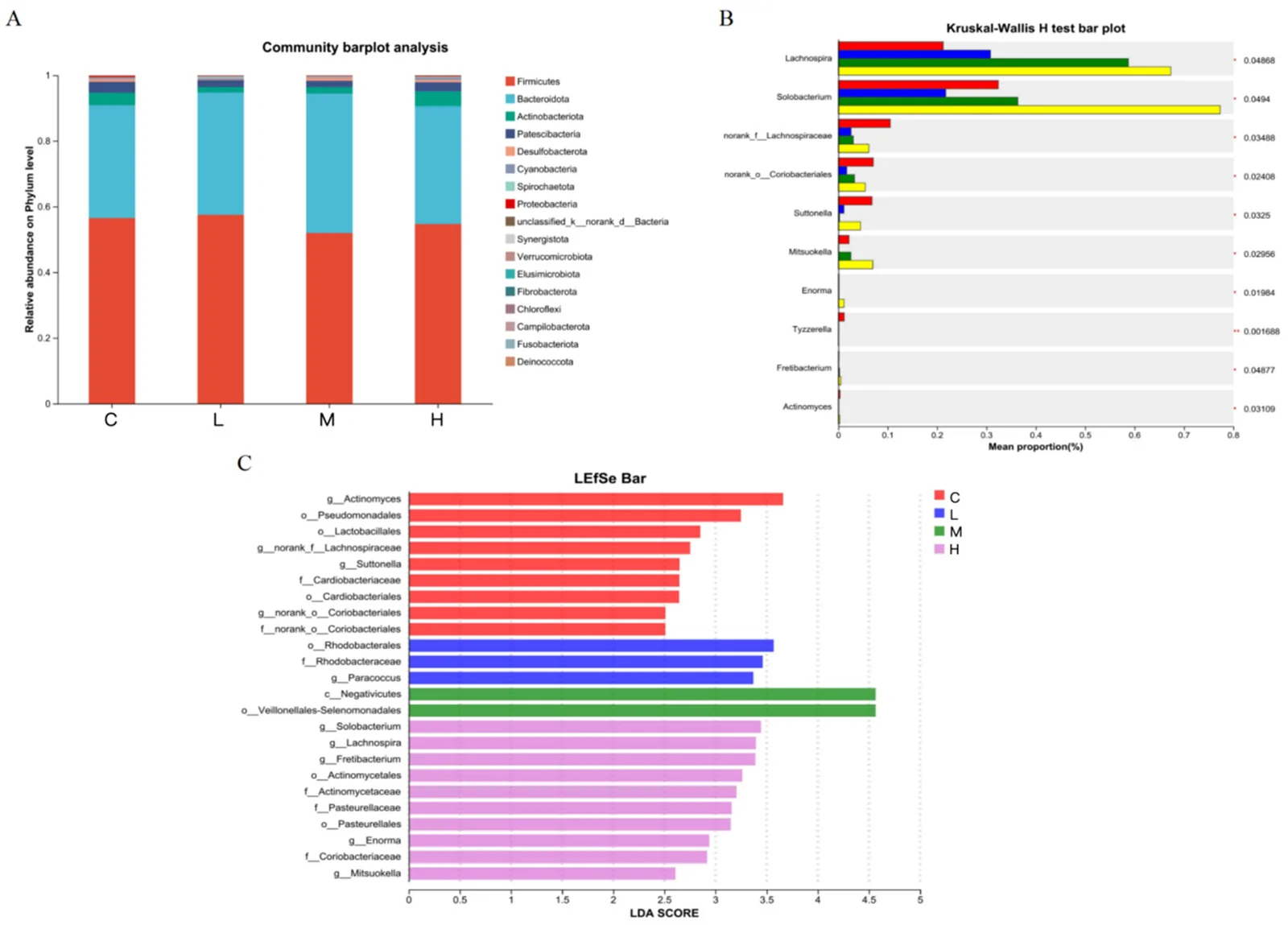
Ruminal microbiota composition of lambs supplemented with *L. salivarius* KS1018. (**A**) Phylum-level composition of ruminal microbiota, (**B**) Kruskal–Wallis test on genus-level composition, (**C**) LEfSe analysis. Red, blue, green, and yellow in (**B**) represent groups C, L, M, and H, respectively. * *p* < 0.05, ** *p* < 0.01.

At the phylum level, *Firmicutes*, *Bacteroidota*, *Actinobacterota*, and *Patescibacteria* were the predominant phyla in the rumen across all groups, representing approximately 80% of the total community abundance (Figure 4A). No significant differences in the relative abundance of these phyla were observed between groups C, L, M, and H.

At the genus-level, Kruskal–Wallis test revealed significant overall variation in the relative abundance of several genera across all experimental groups (Figure 4B). Genera such as *Lachnospira* and *Solobacterium* exhibited significant differences in abundance among groups. Additionally, *Enorma*, *Mitsuokella*, *Tyzzerella*, and *Fretibacterium* also showed statistically significant variation.

LEfSe analysis identified key discriminative genera (LDA score > 2.5) across groups (Figure 4C). Group C was enriched in taxa including *Actinomyces* and *Pseudomonadales*, while group L was dominated by *Rhodobacteraceae* and *Paracoccus*. Group M was enriched with *Negativicutes* and *Veillonellales-Selenomonadales*, whereas group H was enriched in *Solobacterium*, *Lachnospira*, and *Fretibacterium*. Additionally, genera such as *Actinomycetaceae* and *Pasteurellaceae* were present at higher levels in group H.

### 3.4. Metabolic Profiling of the Rumen Fluid Supplemented with L. salivarius KS1018

**Figure 5:**
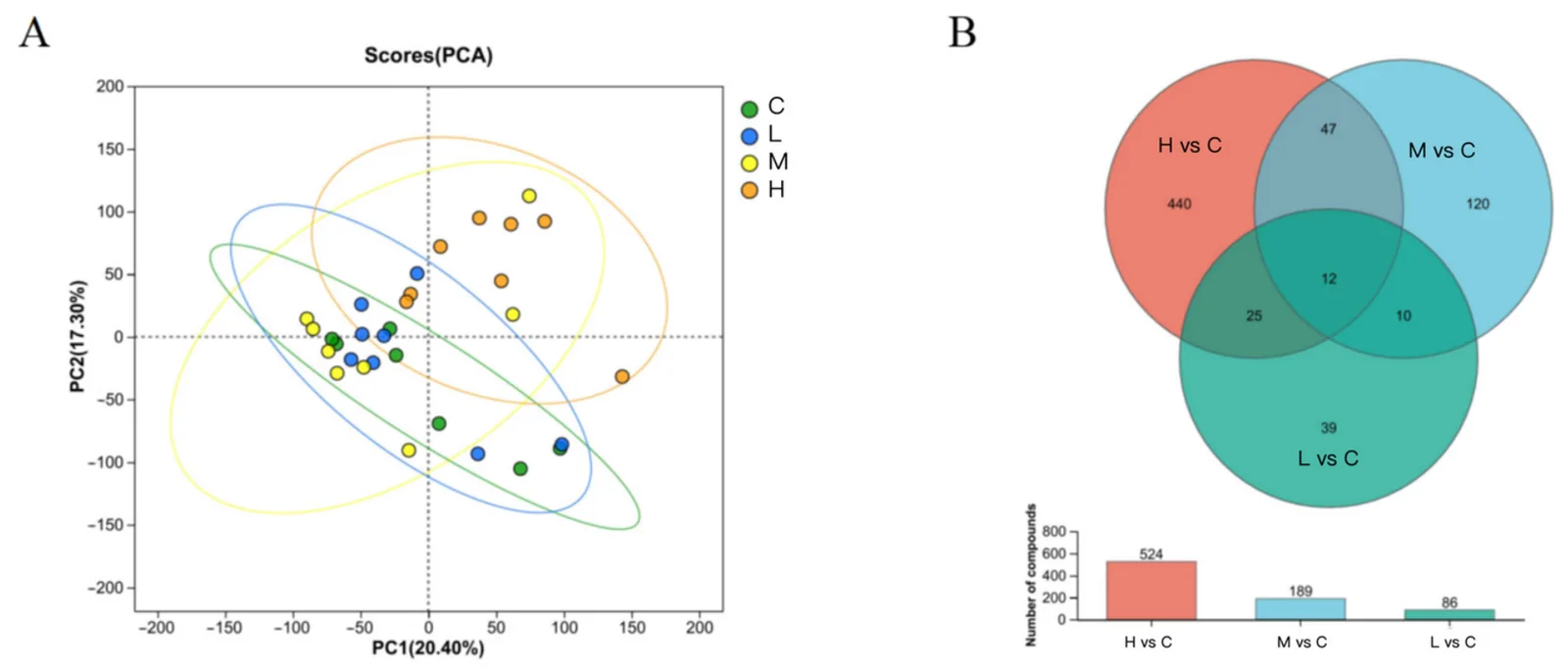
Metabolic profiling of rumen fluid in lambs supplemented with *L. salivarius* KS1018. (**A**) Principal component analysis (PCA), (**B**) venn diagram.

Multivariate analysis suggested dose-related shifts in rumen metabolite profiles following *L. salivarius* KS1018 supplementation. PCA showed partial separation between the control and supplemented groups (Figure 5A), with PC1 and PC2 explaining 20.40% and 17.30% of the variance, respectively. The H samples clustered further from the control relative to group L and M. The Venn diagram showed 524 differential compounds in H, including 440 unique compounds, whereas 189 and 86 differential compounds were observed in M and L, respectively (Figure 5B).

**Figure 6:**
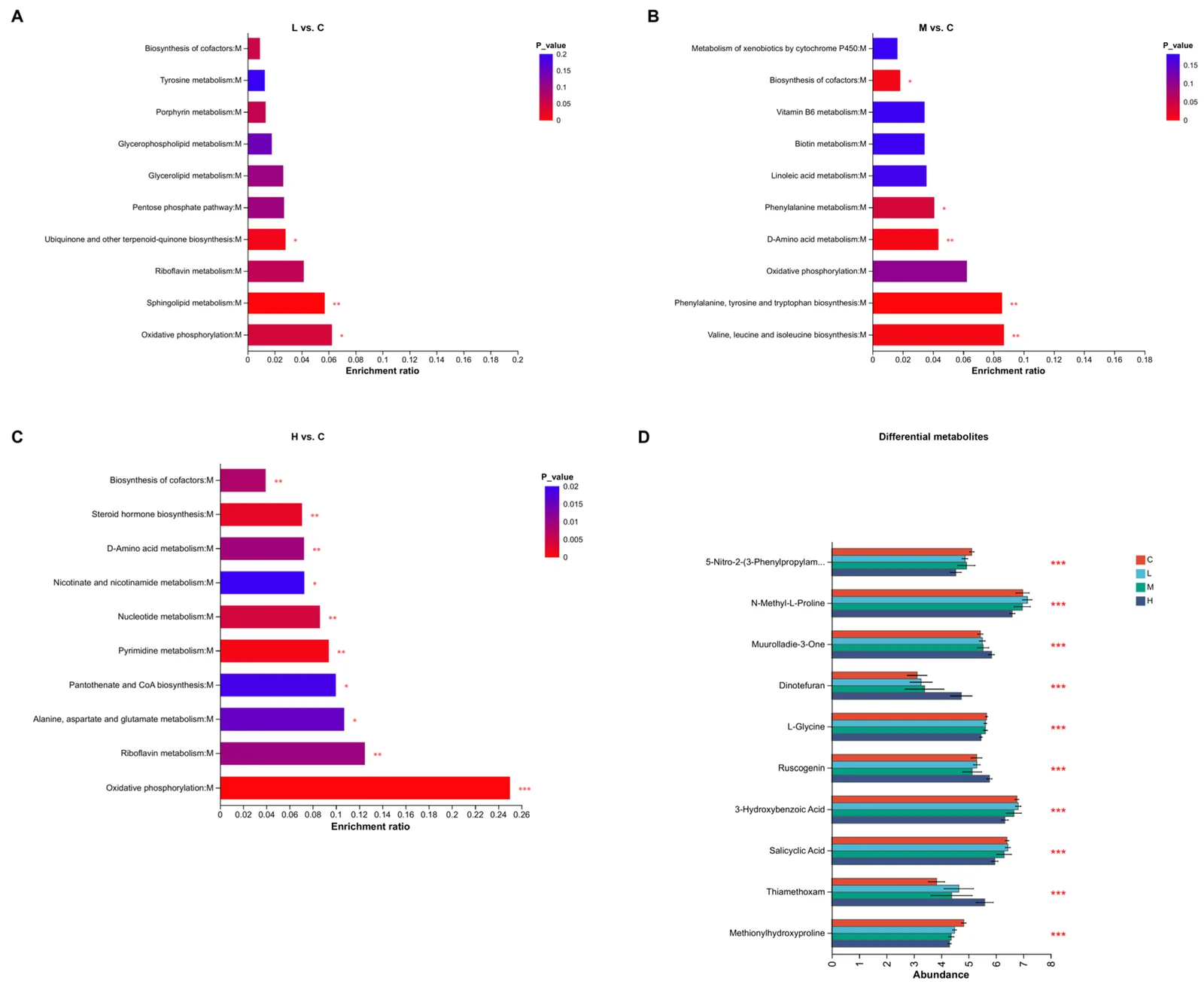
Rumen metabolic pathways enrichment analysis and key differential metabolites between groups in lambs supplemented with *L. salivarius* KS1018. (**A**) KEGG pathway analysis between groups L and C, (**B**) KEGG pathway analysis between groups M and C, (**C**) KEGG pathway analysis between groups H and C, (**D**) ANOVA-selected differential metabolites between groups. * *p* < 0.05, ** *p* < 0.01, *** *p* < 0.001. FDR-adjusted q values are provided in Table S2.

KEGG enrichment analysis focused on metabolism-related pathways with clearer relevance to rumen metabolomic interpretation. Compared with group C, group L showed enrichment of oxidative phosphorylation, sphingolipid metabolism, riboflavin metabolism, and ubiquinone and other terpenoid–quinone biosynthesis (Figure 6A). Group M was mainly characterized by enrichment of valine, leucine and isoleucine biosynthesis; phenylalanine, tyrosine and tryptophan biosynthesis; oxidative phosphorylation; and D-amino acid metabolism (Figure 6B). In group H, oxidative phosphorylation was the most prominently enriched pathway, accompanied by riboflavin metabolism, pyrimidine metabolism, nucleotide metabolism, and D-amino acid metabolism (Figure 6C).

One-way ANOVA identified significant differences in several metabolite features among groups (Figure 6D). These included ruscogenin, muurolladie-3-one, methionylhydroxyproline, salicylic acid, 3-hydroxybenzoic acid, N-methyl-L-proline, and other annotated features. Features putatively annotated as thiamethoxam and dinotefuran also differed among groups and showed their highest abundance in group H. Because these xenobiotic annotations were not confirmed using authentic standards, they were considered tentative and were not used as primary mechanistic evidence. Raw *p*-values and FDR-adjusted q values are provided in Table S2. Detailed annotation information, including fragmentation scores, mass errors, retention times, library identifiers, and QC RSD values, is provided in Table S3.

### 3.5. Rumen Microbiome-Metabolome Association Analysis

**Figure 7:**
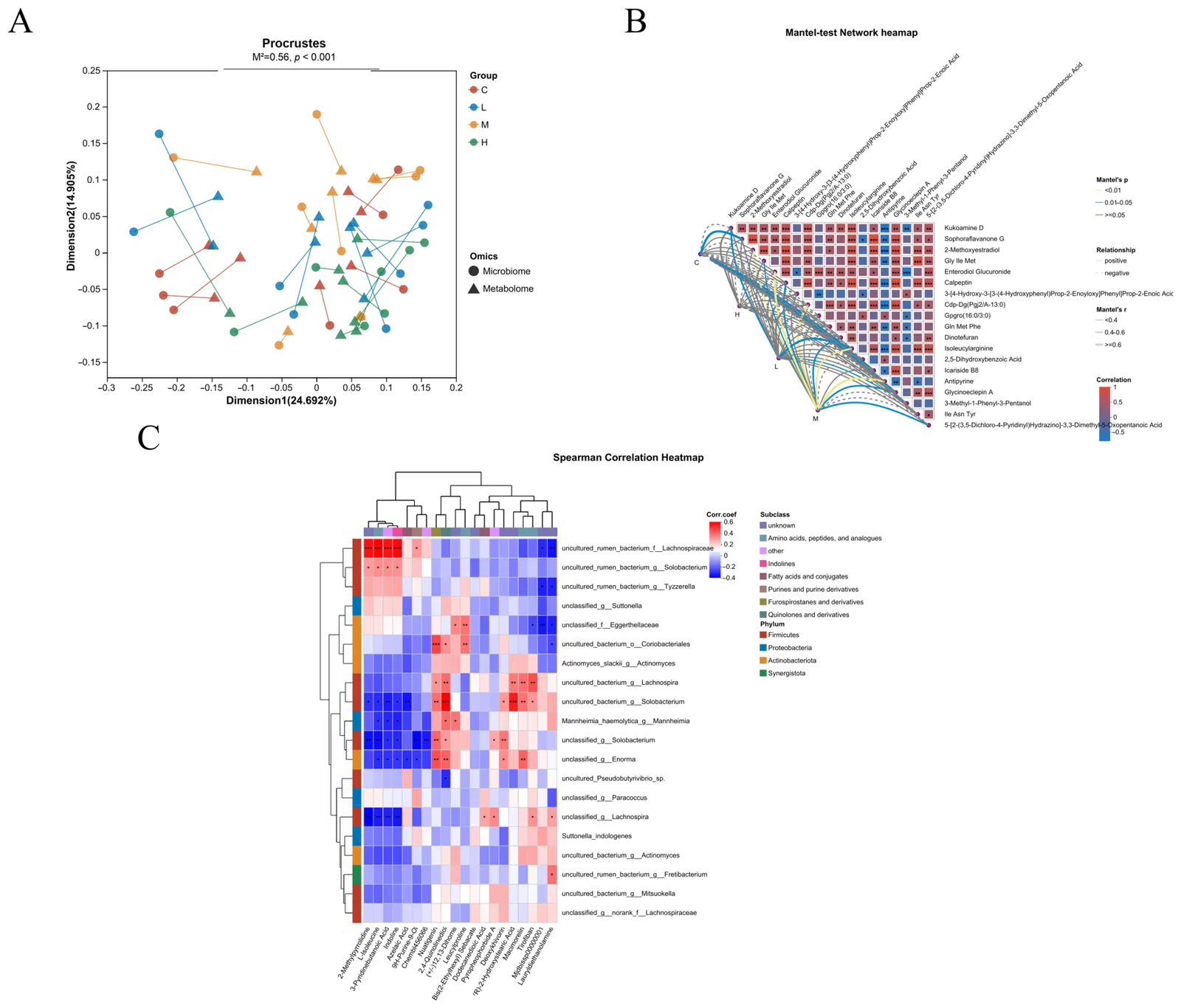
Microbiome–metabolome associations in the rumen of lambs supplemented with *L. salivarius* KS1018. (**A**) Procrustes analysis, (**B**) group-specific Mantel-test network showing associations between genus-level microbial community dissimilarity and individual metabolite distance matrices, (**C**) Spearman correlation analysis of core microbiome and metabolite. * *p* < 0.05, ** *p* < 0.01, *** *p* < 0.001.

The Procrustes analysis (Figure 7A) showed a significant correlation between the microbiome and metabolome datasets (M^2^ = 0.56, *p* < 0.001). The Mantel-test network heatmap (Figure 7B) was used to evaluate associations between microbiome and metabolome profiles across group C, L, M, and H. Mantel’s r-values ranged from −1 to 1, and significant associations were observed between genus-level microbial community dissimilarity and the distance matrices of selected metabolites (*p* < 0.01). In group M, microbial community dissimilarity was strongly correlated with the distance matrices of 2-methoxyestradiol, enterodiol glucuronide, Cdp-Dg(Pgj2/A-13:0), isoleucylarginine, and antipyrine (Mantel’s r ≥ 0.6, *p* < 0.01). In addition, metabolites such as kukoamine D and sophoraflavanone G showed correlation patterns between specific groups (Figure 7B).

Spearman correlation analysis (Figure 7C) identified significant positive correlations between microbial taxa and metabolites. Within Firmicutes, uncultured rumen bacteria affiliated with the family *Lachnospiraceae* and the genus *Solobacterium* were positively correlated with L-isoleucine, 3-pyridinebutanoic acid, indoline, and azelaic acid. Within Actinobacteria, *Actinomyces slackii* and uncultured *Actinomyces* spp. were positively correlated with L-isoleucine, 3-pyridinebutanoic acid, and indoline. Additional correlations included uncultured *Coriobacteriales* with leucylproline, *Mannheimia haemolytica* with 2,4-quinolinediol, unclassified Enorma with nuatigenin and 2,4-quinolinediol, and uncultured *Fretibacterium* spp. with lauryldiethanolamine (Figure 7C).

## 4. Discussion

The present study evaluated the dose-related effects of a rumen-derived *L. salivarius* strain, KS1018, on growth performance and rumen function in Hu lambs under intensive feeding conditions in Xinjiang. Although overall growth responses were limited, *L. salivarius* KS1018 supplementation altered key rumen fermentation readouts, including VFA profiles and ruminal NH_3_-N, and was accompanied by selective genus-level microbial changes and metabolomic profile alterations. Based on this foundation, our study bridges this potential by introducing *L. salivarius* KS1018, a strain originally isolated from the rumen of Duolang sheep, a breed with enhanced fiber degradation capacity, into Hu lamb, a high yielding commercial breed with suboptimal feed efficiency [25]. Notably, this is the first study to integrate rumen fermentation traits with 16S rRNA profiling and metabolomics to evaluate the effect of a Duolang sheep-derived rumen *L. salivarius* strain in Hu lambs, providing a broader view of probiotic associated responses beyond growth metrics alone.

*L. salivarius* KS1018 supplementation did not significantly affect BW trajectories or ADG during the 56-day trial, although serum T3 and T4 concentrations were higher in groups M and H than in group C. Serum GH concentrations did not differ among group C, M, and H, whereas serum T3 and T4 were significantly higher in group M and group H than in the control. Thyroid hormones are tightly linked to whole body energy turnover in ruminants, with T3 as the main biologically active form and T4 serving largely as a prohormone, and their circulating levels are known to shift with nutritional status and physiological load [26]. In ruminant supplementation studies, increases in T3 and T4 have also been reported after adding probiotic containing products, sometimes together with improved performance, for example in finishing lambs receiving a probiotic mixture combined with fibrolytic enzymes, and in lambs supplemented with probiotic combinations [27]. This finding suggests an endocrine response to KS1018 supplementation without a corresponding measurable improvement in short-term growth performance. Therefore, the present results do not establish a production or economic benefit of KS1018 supplementation. Longer trials incorporating individual feed-intake measurements and economic evaluation are required before practical application can be recommended.

The supplementation of Duolang sheep-derived *L. salivarius* KS1018 altered rumen nitrogen metabolism and fermentation profiles in a dose-related manner. Ruminal NH_3_-N concentration was significantly reduced in group H compared with groups C and L, and linear trend analysis further showed a significant decrease in NH_3_-N with increasing KS1018 dose. This suggests that KS1018 supplementation was associated with changes in ruminal nitrogen utilization, as reflected by reduced NH_3_-N concentration. In contrast, total VFA concentration increased significantly in group H and showed a positive linear response to increasing dose, indicating enhanced overall rumen fermentation. Isobutyric acid was also elevated in groups M and H and increased linearly with dose. As a branched-chain VFA, isobutyrate is closely related to microbial amino acid metabolism [28]. However, the lower pH observed in group H compared with group M suggests that high-dose supplementation may shift fermentation balance. Overall, these findings indicate that KS1018 modulated rumen fermentation mainly by reducing NH_3_-N and increasing total VFA and isobutyric acid concentrations, while also highlighting the importance of dose optimization.

The ruminal microbiota analysis revealed that *L. salivarius* KS1018 supplementation did not significantly affect alpha diversity indices, indicating preserved microbial richness and evenness across groups. Beta diversity analysis (PCoA) demonstrated overlapping community structures, suggesting minimal compositional divergence despite dose-related shifts in specific genera. The consistent dominance of *Firmicutes*, *Bacteroidota*, *Actinobacterota*, and *Patescibacteria* across all groups, together with unchanged alpha diversity and overlapping PCoA clusters, suggests that the overall taxonomic structure of the rumen microbiota remained broadly stable after KS1018 supplementation. Despite the overall stability in community structure, *L. salivarius* KS1018 supplementation was associated with selective changes in the relative abundance of several genera. In group H, the significant increase in *Lachnospira* aligns with its known role in carbohydrate fermentation and SCFA production which is essential for rumen epithelial energy supply [29]. This genus-level increase coincided with higher total VFA concentrations observed in group H. However, the present data do not support a direct link between *Lachnospira* abundance and butyrate concentration, because butyrate did not significantly increase among treatments. Therefore, this association should be interpreted cautiously. Additionally, the enrichment of *Negativicutes* and *Veillonellales-Selenomonadales* in group M is consistent with their documented ability to metabolize lactate into propionate, a process that has been linked to lactate utilization and propionate production and support gluconeogenesis [30]. The increase in *Solobacterium* in group H may be associated with sulfur-containing amino acid metabolism, but functional implications such as H_2_S production require further validation [31]. The observed genus-level shifts, particularly those associated with improved fiber fermentation and nitrogen-related metabolism in ruminants, resemble microbial adaptations identified in Duolang sheep, suggesting that donor-derived strains may be associated with selected rumen microbial and fermentation responses, although functional transfer requires direct validation.

Metabolomic profiling indicated that KS1018 supplementation was associated with dose-related differences in metabolism-related rumen pathways. In group H, enrichment of oxidative phosphorylation, pyrimidine metabolism, and nucleotide metabolism occurred alongside increased total VFA concentrations, suggesting possible changes in energy metabolism and nucleotide turnover. In group M, enrichment of valine, leucine and isoleucine biosynthesis and phenylalanine, tyrosine and tryptophan biosynthesis occurred alongside increased isobutyrate concentrations, suggesting possible involvement of microbial amino acid metabolism. Riboflavin metabolism was also enriched in supplemented groups, which may be related to microbial cofactor metabolism because riboflavin serves as a precursor of FMN and FAD and has been associated with rumen fermentation in Hu lambs [32,33]; however, this interpretation requires further validation. Overall, the biologically relevant enrichment patterns were mainly related to energy, nucleotide, amino acid, and cofactor metabolism. In addition, features putatively annotated as thiamethoxam and dinotefuran correspond to neonicotinoid xenobiotics rather than endogenous rumen metabolites. Their detection may reflect trace exposure from feed or the environment, or tentative annotation in the untargeted LC-MS workflow. Because these annotations were not confirmed using authentic standards, they were interpreted cautiously and were not used as mechanistic evidence for KS1018-induced metabolic regulation.

Procrustes and Mantel-test analyses indicated significant correspondence between the rumen microbiome and metabolome in Hu lambs supplemented with KS1018, supporting an association between microbial community structure and ruminal metabolic output. Similar microbiome–metabolome linkages have been reported in integrated rumen multi-omics studies in sheep and cattle, in which microbial composition was significantly associated with metabolite profiles and fermentation-related traits [34,35]. In the present study, this correspondence was accompanied by genus-level shifts, including enrichment of *Lachnospira* and *Negativicutes*, together with dose-related differences in metabolism-related pathways, such as oxidative phosphorylation and nucleotide metabolism in group H and amino acid biosynthesis in group M. Spearman correlation and network analyses further showed that *Firmicutes* taxa, including *Lachnospiraceae* affiliated features and *Solobacterium*, showed positive correlations with nitrogen-related metabolites such as L-isoleucine and isoleucylarginine, consistent with the enrichment of branched-chain amino acid biosynthesis pathways. Higher isobutyrate in the supplemented groups was also linked to these branched-chain amino acid-related metabolic patterns. Together, these data suggested that Duolang sheep-derived *L. salivarius* KS1018 may be associated with coordinated changes in selected microbial taxa and rumen metabolites, providing hypotheses for future functional validation. A limitation of this study is that one lamb in group M developed ORC and received therapeutic treatment, including antibiotics. Although this lamb was excluded from growth-performance analysis, it remained included in endpoint serum hormone, rumen fermentation, microbiome, and metabolomics analyses. Therefore, possible effects of ORC and antibiotic treatment on these endpoint outcomes cannot be fully excluded.

## 5. Conclusions

This study evaluated the dose-related effects of the rumen-derived *L. salivarius* KS1018 strain on rumen fermentation, microbiota, and metabolomic profiles in Hu lambs. KS1018 supplementation was associated with altered rumen fermentation, including lower NH_3_-N concentration and higher total VFA and isobutyrate concentrations at higher doses. Overall microbial diversity and community structure remained broadly stable, whereas selected genus-level differences and metabolism-related metabolomic shifts were observed. KEGG enrichment mainly indicated changes related to energy, nucleotide, amino acid, and cofactor metabolism. These findings suggest that KS1018 may modulate rumen fermentation and metabolic profiles in lambs, but longer-term studies with individual feed-intake and production measurements are needed to confirm practical benefits and optimize dosage.

## Figures and Tables

**Table 1:** Growth performance of lambs supplemented with *L. salivarius* during a 56-day trial.

Item	Group				*p*-Value
	C	L	M	H	
Initial BW (kg)	32.29 ± 1.50	33.36 ± 1.22	32.33 ± 3.16	30.88 ± 2.71	/
Day 28 BW (kg)	41.99 ± 2.98	43.12 ± 1.30	42.00 ± 3.49	39.89 ± 4.86	/
Day 56 BW (kg)	48.51 ± 4.70	48.35 ± 3.00	48.94 ± 4.13	47.12 ± 4.62	/
Overall ADG (g)	289.80 ± 68.49	267.63 ± 52.94	296.68 ± 52.25	290.18 ± 39.82	0.733
Day 1–28 ADG (g)	346.43 ± 74.35	348.66 ± 36.32	345.41 ± 47.32	321.88 ± 130.91	0.907
Day 29–56 ADG (g)	233.16 ± 64.57	186.61 ± 87.46	247.96 ± 70.68	258.48 ± 106.32	0.366
ADFI (g)	1600.00	1562.50	1545.83	1645.83	/
FCR	5.52	5.84	5.21	5.67	/

Note: Repeated BW measurements were analyzed using a linear mixed-effects model, whereas ADG was analyzed using ordinary one-way ANOVA.

**Table 2:** Dose-related effects of *L. salivarius* KS1018 supplementation on rumen fermentation parameters in Hu lambs during a 56-day trial.

Item	Group				*p*-Value	*p*-Linear
	C	L	M	H		
pH	5.90 ± 0.36	5.78 ± 0.27	6.11 ± 0.63	5.49 ± 0.39	0.055	0.216
Ammonia Nitrogen (mg/100 mL)	39.96 ± 5.06	41.78 ± 4.10	31.34 ± 8.04	30.11 ± 7.07	0.001	<0.001
Acetic Acid (mg/kg)	18,350.27 ± 3127.12	20,608.11 ± 3477.65	16,854.75 ± 3694.64	22,025.05 ± 5300.37	0.072	0.277
Propionic Acid (mg/kg)	2417.29 ± 560.07	2846.01 ± 1278.26	2332.43 ± 1660.81	3458.95 ± 1299.69	0.306	0.223
Butyric Acid (mg/kg)	1449.97 ± 543.29	1177.48 ± 403.04	855.07 ± 351.20	1244.93 ± 507.55	0.107	0.217
Isobutyric Acid (mg/kg)	885.43 ± 911.32	1559.51 ± 1956.49	3376.17 ± 2245.04	4359.31 ± 434.92	<0.001	<0.001
Isocaproic Acid (mg/kg)	575.55 ± 185.98	535.13 ± 545.98	780.53 ± 696.26	521.28 ± 541.11	0.751	0.925
Total VFA (mg/kg)	23,678.53 ± 3864.21	26,726.25 ± 4967.64	24,198.95 ± 5712.95	31,609.53 ± 6712.53	0.031	0.024

## Data Availability

The sequencing data are available in the NCBI Sequence Read Archive (SRA) under accession number PRJNA1284226, and the metabolomics data are available in MetaboLights under accession number MTBLS12659.

## Supplementary Materials

The following supporting information can be downloaded at: https://www.mdpi.com/article/10.3390/microorganisms14081641/s1, Table S1: Nutrient and composition analysis of feed samples for Hu lambs; Table S2: Candidate differential metabolite features identified by multi-group comparisons, including group means, VIP scores, raw *p*-values, and FDR-adjusted q values; Table S3: Annotation information and confidence metrics for detected metabolite features.

## Abbreviations

CFU	Colony-forming units
VFA(s)	Volatile fatty acid(s)
NH_3_-N	Ammonia nitrogen
SCFA(s)	Short-chain fatty acid(s)
BW	Body weight
ADG	Average daily gain
ADFI	Average daily feed intake
FCR	Feed conversion ratio
T3	Triiodothyronine
T4	Thyroxine
GH	Growth hormone
