# Supplementary material for: Integrative Microbiome–Metabolome Analysis Reveals Dose-Related Effects of Rumen-Derived Ligilactobacillus salivarius KS1018 on Rumen Function in Lambs
Source: Microorganisms. 2026 Jul 27;14(8):1641. doi: 10.3390/microorganisms14081641 (PMC13515492; doi:10.3390/microorganisms14081641)
Supplement: Supplementary file 1 [file microorganisms-14-01641-s001.zip › Table S1. Nutrient and Composition Analysis of Feed Sample on Hu lambs.pdf]

Table S1 Nutrient and Composition Analysis of Feed Sample on Hu lambs

Ingredient	Result	Unit
Iodine	0.35	mg/kg
Iron (Fe)	180.84	mg/kg
Manganese (Mn)	35.08	mg/kg
Copper (Cu)	21.95	mg/kg
Zinc (Zn)	305.29	mg/kg
Calcium content	0.50	%
Total phosphorus content	0.30	%
Acid detergent fiber (ADF)	33.16	%
Neutral detergent fiber (NDF)	37.28	%
Vitamin A	0.10	mg/kg
Vitamin D3	Not detected	--
Vitamin E	7.61	mg/kg
Niacin	5.24	mg/kg
Ether extract	1.94	%
Non-fibrous carbohydrates	29.56	%
Protein	13.54	%
Dry matter	90.04	%
